# Improving patient understanding of oncology imaging: radiologist and patient evaluation of summarised versus full-length AI-simplified reports from a tertiary cancer centre

**DOI:** 10.1186/s40644-026-01031-x

**Published:** 2026-04-13

**Authors:** Ana S. F. Ribeiro, Olga Husson, Sheila Matharu, Saffron Cox, Davide Meo, Richard Sidebottom, Robby Emsley, Karen Thomas, Joshua Shur, Francesca Castagnoli, Sharmin Malekout, Elizabeth Robinson, Chin Lian Ng, Christian Kelly-Morland, Wim J.G. Oyen, Winette T. A. van der Graaf, Christina Messiou

**Affiliations:** 1 Department of Radiology and Radiology Research and Artificial Intelligence Hub, The Royal Marsden NHS Foundation Trust, Downs Road, Sutton, London, SM2 5PT UK; 2 Division of Radiotherapy and Imaging, The Institute of Cancer Research, 15 Cotswold Road, Sutton, London, SM2 5NH UK; 3 Department of Medical Oncology, Erasmus MC Cancer Institute, Erasmus University Medical Centre, PO Box 5201, Rotterdam, AE 3008 The Netherlands; 4 Department of Public Health, Erasmus University Medical Centre, PO Box 2040, Rotterdam, CA 3000 The Netherlands; 5 Department of Surgical Oncology, Erasmus MC Cancer Institute, Erasmus University Medical Centre, PO Box 5201, Rotterdam, AE 3008 The Netherlands; 6 Department of Medical Oncology, Netherlands Cancer Institute, PO Box 90203, Amsterdam, BE 1006 The Netherlands; 7 Department of Research Data and Statistics Unit, The Royal Marsden NHS Foundation Trust, Downs Road, Sutton, London, SM2 5PT UK; 8 Department of Biomedical Sciences, Department of Nuclear Medicine, Humanitas University and Humanitas Clinical and Research Centre, Via Alessandro Manzoni, 56, 20089 Rozzano, MI Italy; 9 Department of Radiology and Nuclear Medicine, Rijnstate Hospital, PO Box 9555, Arnhem, TA 6800 The Netherlands; 10 Department of Radiology and Nuclear Medicine, Radboud University Medical Centre, PO Box 9101, Nijmegen, 6500 The Netherlands

**Keywords:** Simplified imaging reports, Artificial intelligence, Large language models, Patient preferences

## Abstract

**Background:**

Oncology practice is increasingly aiming to be patient centric. Imaging is a decisive part of the management of cancer patients and with the introduction of Digital Health Records (DHR) patients have the possibility of accessing their imaging results independently, yet the optimal way of doing so is still not clear. The introduction of Large Language Models (LLM) offers the potential to turn radiology reports into a clearer, accessible and unambiguous format and to democratise patient’s access to their own medical records.

**Methods:**

A multi-reader retrospective Service Evaluation (SE) conducted at a tertiary oncology hospital aimed to assess the capability of an LLM to generate two versions of simplified oncology imaging reports. The SE assessed Patient and Public Involvement (PPI) representatives and healthcare professionals’ (HCP) preferences using original radiology reports from two cohorts, colorectal (*n* = 30) and lung (*n* = 30) cancer. A Prompt-development phase created two prompts to generate the summarised (version A) and the full-length (version B) report versions. The review was performed by radiologists with 360 reads and PPI representatives with 180 reads.

**Results:**

Radiologists scores between summaries and full-length reports differed per cohort. In the lung cohort, version A was rated higher for factual correctness (*P* = 0.001), completeness (*P* < 0.0001), accessibility and readability (*P* = 0.026), and benefit to patients (*P* < 0.0001). The opposite was seen in the colorectal cohort, version B achieved consistently higher scores (*P* < 0.002). When the two cohorts were combined, median scores for version A and B did not differ significantly (all *P* > 0.057). PPI reviews indicated that full-length reports were favoured significantly (*P* < 0.0001). Qualitative results from radiologists and PPI identified incorrect statements (*n* = 28), complex terminology (*n* = 18), addition of confusion (*n* = 10), and missing information (*n* = 10).

**Conclusions:**

LLM simplified reports have the potential to improve patient accessibility in oncology imaging. PPI and HCP preferences for summarised versus full-length reports vary. Findings suggest these outputs are likely to benefit from appropriate adjustments to individual patient needs and clinical context. Reports with incorrect, confusing and missing content, highlight that LLM need improvement, ahead of potential clinical use in this setting.

**Supplementary Information:**

The online version contains supplementary material available at 10.1186/s40644-026-01031-x.

## Background

Imaging is a central tenant of oncological care, playing a critical role in detection, diagnosis, monitoring and assessment of response. Patients undergo numerous scans, from diagnosis to follow-up, as part of routine clinical practice or assessing the efficacy of medical treatment. Radiology reports are therefore a critical element of cancer patient management and medical notes [1]. The current healthcare model is based on a multidisciplinary approach, which has seen a significant improvement and emphasis on patient engagement and empowerment for patients to become active participants in their health management [2].

With the wide adoption of Digital Health Records (DHR), there is a real opportunity to improve patient engagement in imaging oncology [3]. DHR enable patients to independently access their health records through digital platforms. By ensuring that imaging records are available in a format that patients themselves can clearly understand, patients can better engage in shared decision-making and be active participants in their treatment pathway [4, 5].

Democratising patients’ access to their own medical records is not a new concept [6–8]. However, barriers such as the manpower needed to provide accurate and nuanced translations have not yet made this into a widely adopted clinical reality. The introduction of Artificial Intelligence (AI) Large Language Models (LLM) presents a possible solution to translate unstructured clinical reports into a structured, tailored and patient-friendly report. LLM have the potential to enhance patient communication for medical records, including imaging reports [9] by improving the clarity, accessibility and unambiguousness of the information presented. This technology can transform complex medical jargon and technical terminology into plain language, making the imaging reports more accessible to patients [10]. Similar applications have been explored, in the context of AI chatbot assistants [11], structuring imaging reports [12], and the conduct of radiology board examinations [13]. This may be particularly valuable in oncology, where imaging reports often contain complex terminology that can be challenging for patients to understand. Furthermore, patients are often required to undergo multiple scans over the course of their treatment, and a better understanding of the findings may help to align the patients with their management plan.

This Service Evaluation (SE) aimed to evaluate the potential of a LLM to generate simplified oncology imaging reports by comparing two prompt-generated outputs, a summarised (Prompt A) and a full-length (Prompt B) version of Computed Tomography scan (CT scan) reports from colorectal and lung cancer patients. The SE sought to assess the accuracy, completeness, and clinical relevance of the LLM-simplified reports from the perspective of radiologists, and to explore Patient and Public Involvement (PPI) representatives’ preferences regarding accessibility, readability, and preference for each report version.

## Methods

A Service Evaluation (SE) conducted at The Royal Marsden NHS Foundation Trust (RM), a specialist tertiary oncology centre in the United Kingdom, aimed to compare two prompt-generated outputs from Open.AI GPT-4 deployed in the RM Trust secure Azure environment.

The SE was approved by the Committee for Clinical Research (CCR) - Royal Marsden NHS Foundation Trust on the 26th of February 2024. An amendment submitted to include a PPI reader sub-study was approved on the 30th of June 2025.

All data was subject to the National Data Opt-Out (NDOO) mesh framework and fully anonymised by the RM Trust Performance and Information team and the study was conducted in accordance with the Declaration of Helsinki. Approval was also received from the RM Trust Information Governance board following a Data Protection Impact Assessment approval.

Imaging reports were prepared using Microsoft Excel for Microsoft 365, Version 2505 and shared using private channels with reviewers and readers. GraphPad Prism (Version 10) was used for quantitative data analysis. In addition, readers were able to provide open comments, analysed using a qualitative descriptive approach, to provide contextual insights complementing the quantitative data.

The Prompt-development phase took place between March 2024 and March 2025. The reader phase was performed using retrospective CT scans from deceased colorectal and lung cancer patients, which had been performed between 2015 and 2016. Radiologist review was performed between May 2025 and July 2025, and PPI representatives review between July 2025 and August 2025.

### Service evaluation design

This retrospective single centre, multi-reader service evaluation was divided into two phases: (i) prompt development phase and (ii) multi-reader phase. Formal sample size was not calculated, tumour types were chosen pragmatically to be representative across gender, CT scans were used as they are a frequent imaging modality in oncology, and number of readers and LLM was selected based on availability of internal readers and resources.

### Prompt-development phase

Four reviewers, consisting of three experienced radiographers (AR, RE, DM) and one experienced radiologist (RS), together with two senior data analysts (SM, SC), performed the prompt development over five steps, with 13 selected reports used for this phase only. Two prompts using the chosen model Open.AI GPT-4 were developed. Prompt A – summary of the original report and Prompt B – full length simplification of the original report.

Levels of overall agreement between the original report and the output (report generated by the prompt) were performed using a 5-point Likert scale (1 = strongly disagree, 2 = disagree, 3 = neutral, 4 = agree, 5 = strongly agree).

The Prompt-development engineering attempted to reduce bias by following a stepwise methodology as per Table 1 (also see Additional file 1. Prompt development steps), with a minimum of two reviewers per step.

Each reviewer scored a total of 20 outputs each for Prompt A and Prompt B. The results gave good repeatability and the selected prompts were:

Prompt A (three shot) – summary of the original report:

“Generate a clear and succinct summary of this medical imaging report in professional language. Include important findings, potential diagnoses, and any suggested next steps, framed in a way that can be easily explained to a patient. Here are three examples of how the summary should be generated: Example 1; Example 2; Example 3. Text to be summarised: “.

Prompt B (zero shot) – full length simplification of the original report:

“Rewrite this radiology report in clear, professional, simple language that a patient with no medical background can easily understand. Keep the structure of the original report and where applicable use the same headings like for example: ‘Background’, ‘Clinical History’, ‘Findings’, ‘Opinion’, ‘Conclusion’, and so on. Provide an accurate, third-person explanation of the findings and their meaning. Avoid medical jargon, repetition, and ensure the tone remains professional. Do not address the patient directly. Report:”

**Table 1:** Prompt development (also refer to Additional file 1. Prompt development steps)

Steps	Number of readers	Number of prompts	Selected prompt(s) A	Selected prompt(s) B	Process	Hyperparameters
**1**	2	30	2; 6	16; 28	Initial prompt development: Score based selection of the best four prompts for A and B.	temperature=0 max_tokens=1600 top_p = 1
**2**	3	4	6	28	Previously selected prompts used to generate outputs using new report; Score based selection of the best prompts for A and B.	temperature=0 max_tokens=1600 top_p = 1
**3**	4	10	6.1 (variation 1 from report 6)	28.5 (variation 5 from report 28)	Five variations for each prompt are developed iteratively, taking into account: Prompt 6 (A): (i) keep healthcare professional tone; (ii) write in 3rd person; (iii) avoid repetition by engineering the prompt wording; iv) < 2 paragraphs. Prompt 28 (B): (i) keep healthcare professional tone; (ii) write in 3rd person; (iii) avoid repetition by engineering the prompt wording; (iv) keep similar structure to original report.	temperature=0 max_tokens=1600 top_p = 1
**4**	4	4	6.1 (three shot)	28.5 (zero shot)	Three additional report examples are used to create variations of the previously selected prompts: three_shot zero_shot.	temperature=0 max_tokens=1600 top_p = 1
**5**	4	2	6.1 (three shot)	28.5 (zero shot)	Repeatability step for prompts A and B; run ten times each and scored to ensure model confidence with the set hyperparameters.	temperature=0 frequency_penalty=0 presence_penalty=0 top_p = 1

### Multi-reader phase

The multi-reader phase consisted of a radiologist review, with three experienced radiologists per cohort (lung cohort: ER; CLN; CKM and colorectal cohort: JS; FC; SMT). The PPI review was performed by three representatives (DK; RG; ML), with previous experience of oncology imaging research, who reviewed both lung and colorectal cohorts. All readers reviewed files containing the original report, together with versions A and B simultaneously. Levels of agreement for output scoring against five categories were performed using a 5-point Likert scale (1 = strongly disagree, 2 = disagree, 3 = neutral, 4 = agree, 5 = strongly agree) for versions A and B.

### Radiologist review

Colorectal and lung cancer cohorts each contained 30 original reports. Prompts A and B were used once to generate 30 version A and 30 version B reports per cohort, with a total of 120 generated reports across colorectal and lung cohorts. An example for colorectal and lung reports, containing the original report and versions A and B can be seen in the additional file 2. Three radiologists per cohort performed a total of 360 reads, as per Table 2. Following a washout period of > 6 weeks, an additional randomly selected 6 cases for repeatability were scored per radiologist, with a total of 72 reads.

Categories selected were based on earlier studies [14, 15], to include: (i) Factual correctness; (ii) Completeness; (iii) Potential harm; (iv) Accessibility and Readability; and (v) Benefit to patients.

### PPI review

A subset of 10 reports where radiologist scores were > 4 for factual correctness for both version A and B were selected for the PPI review. Three PPI readers performed a total of 180 reads for colorectal and lung cohorts, which also included reads for the original reports alongside versions A and B. Categories were: (i) Accessibility and Readability; (ii) Benefit to patients and (iii) Preferred report version.

**Table 2:** Multi reader study design for Radiologist and PPI review

Review	Cohort	Readers	Original reports	Version A reports (A)	Version B reports (B)	Repeatability subset	Total reads per cohort
Radiologists	Colorectal	*n* = 3	*n* = 30	*n* = 30 3 × (30_A_)	*n* = 30 3 × (30_B_)	*n* = 6 3 × (6_A_ + 6_B_)	*n* = 180 *n* = 36 (repeatability)
	Lung	*n* = 3	*n* = 30	*n* = 30 3 × (30_A_)	*n* = 30 3 × (30_B_)	*n* = 6 3 × (6_A_ + 6_B_)	*n* = 180 *n* = 36 (repeatability)
PPI	Colorectal	*n* = 3	*n* = 10 3 × (10)	*n* = 10 3 × (10_A_)	*n* = 10 3 × (10_B_)	N/A	*n* = 90
	Lung	*n* = 3	*n* = 10 3 × (10)	*n* = 10 3 × (10_A_)	*n* = 10 3 × (10_B_)	N/A	*n* = 90

### Data analysis

#### Quantitative analysis

Ordinal data were analysed using descriptive statistics with medians and 25% and 75% interquartile ranges (IQR) reported to summarise central tendency. The radiologist review included median differences (version B – version A) and for statistical comparisons between version A and version B, Wilcoxon signed-rank test was applied. This non-parametric paired test was chosen as it does not assume normal distribution and is appropriate for repeated measures within the same set of cases across versions. A *P*-value < 0.05 was considered statistically significant and no correction was done for multiple comparisons. Percentage scores were also calculated as a useful summary for interpretation, for Q1, Q2, Q4, Q5 > 4 and Q3 ≤2.

The PPI review consisted of comparing scores across the three report versions (original, version A and version B), by applying the Friedman test. Post-hoc Wilcoxon signed-rank tests were performed to assess pairwise differences between version A and version B. Median differences, Wilcoxon signed-rank test statistics (*W*), and associated *P*-values were reported, with statistical significance set at *P* < 0.05 and no correction for multiple comparisons.

#### Qualitative analysis

Open comments from readers were analysed through an inductive coding process, a bottom-up approach that highlights nuances and enriches the quantitative data in exploratory studies [16]. All comments were reviewed in full and systematically coded by main researcher AR and independently checked by researcher OH.

## Results

### Radiologist review

**Fig. 1:**
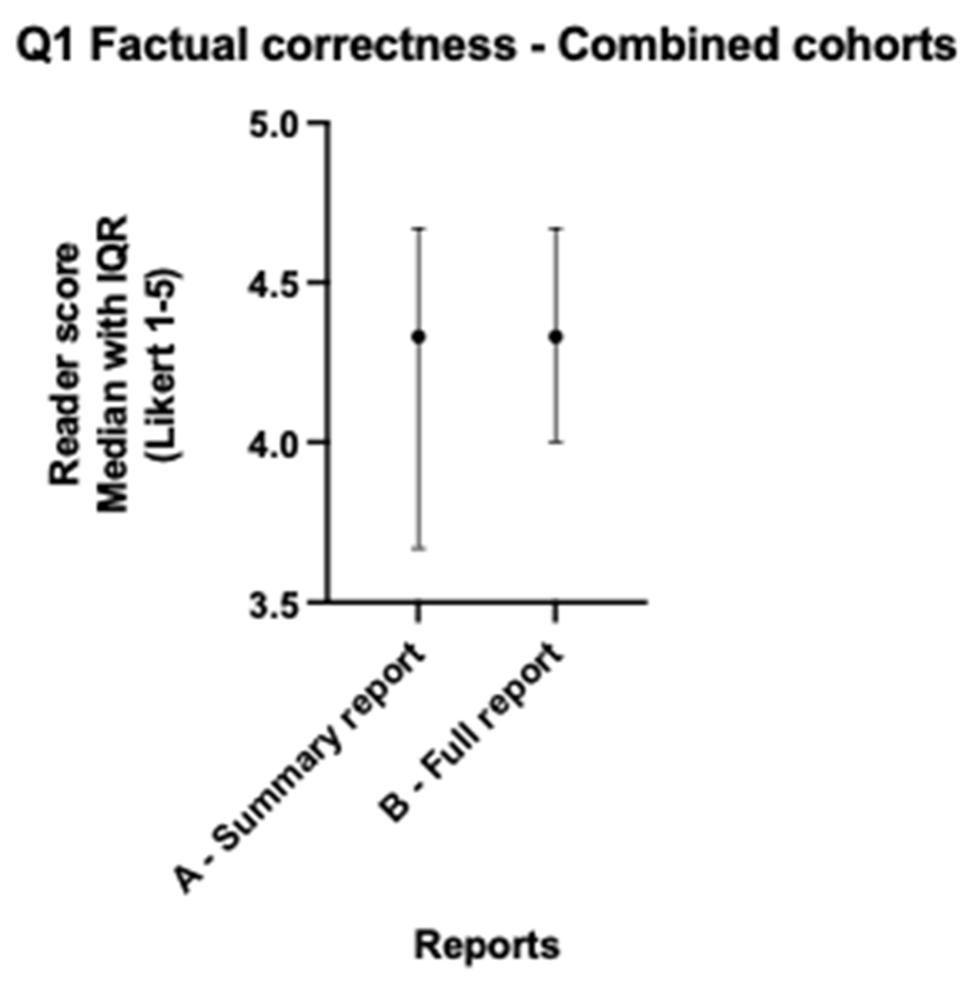
Radiologist colorectal & lung - Factual correctness median scores

**Fig. 2:**
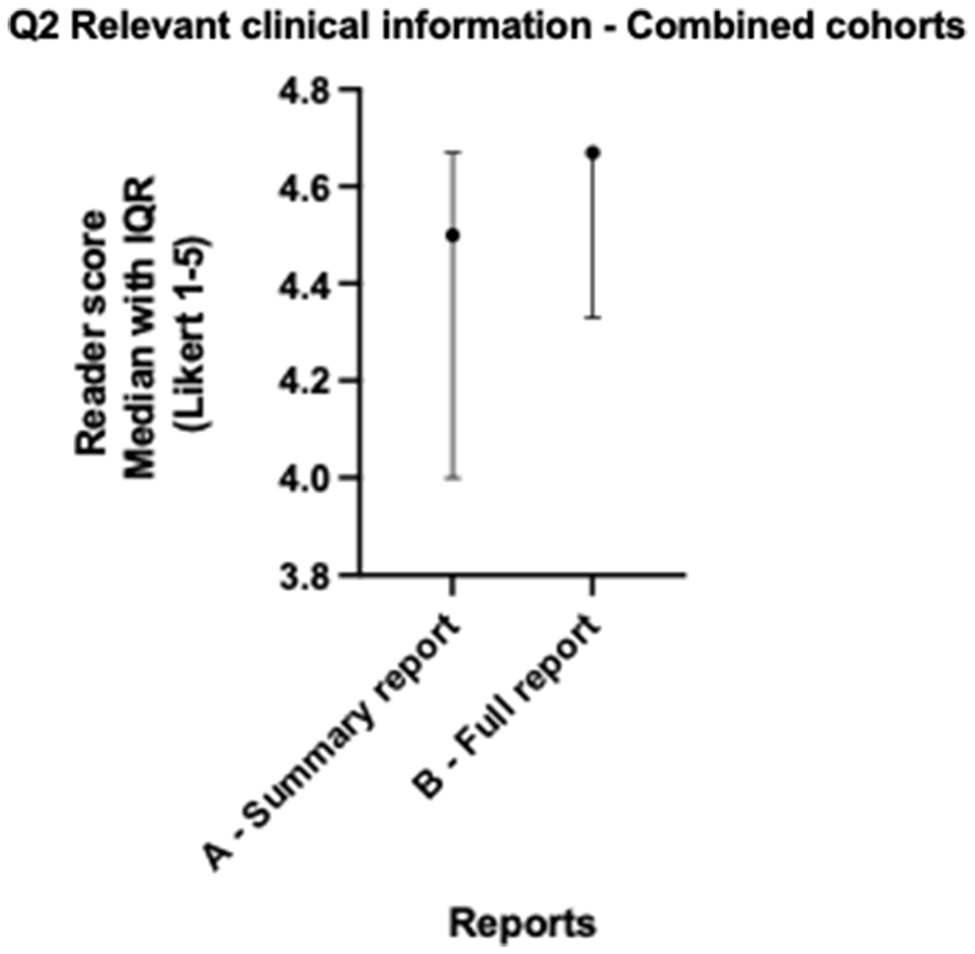
Radiologist colorectal & lung - Relevant clinical information median scores

**Fig. 3:**
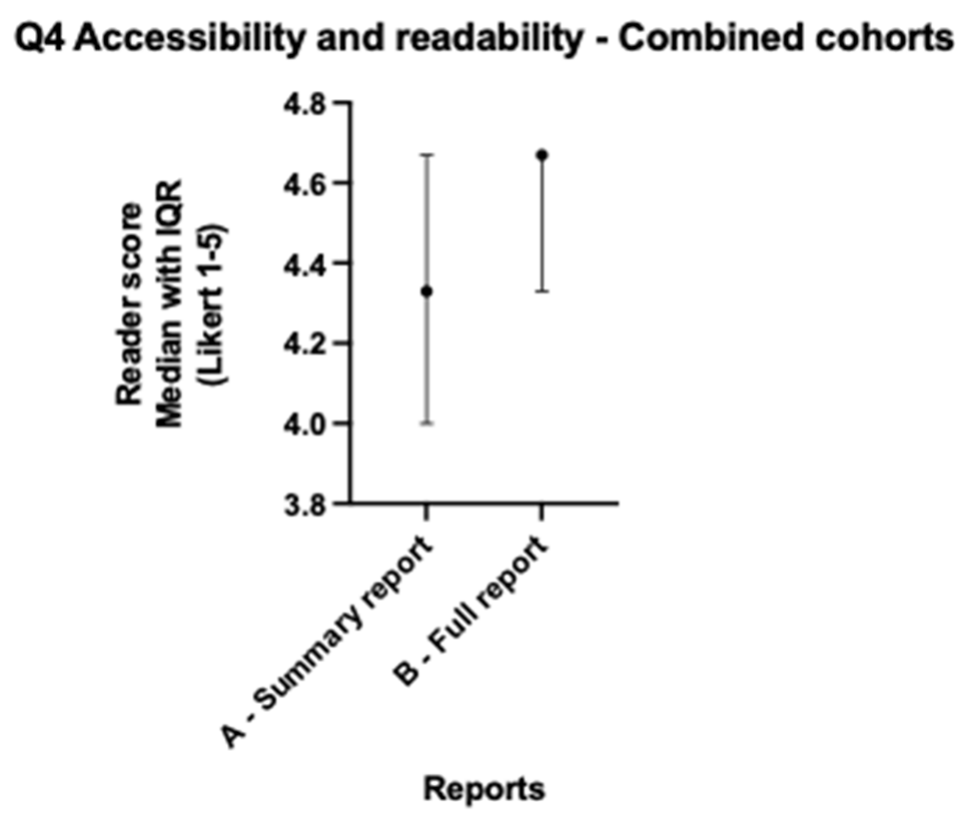
Radiologist colorectal & lung - Accessibility and readability median scores

**Fig. 4:**
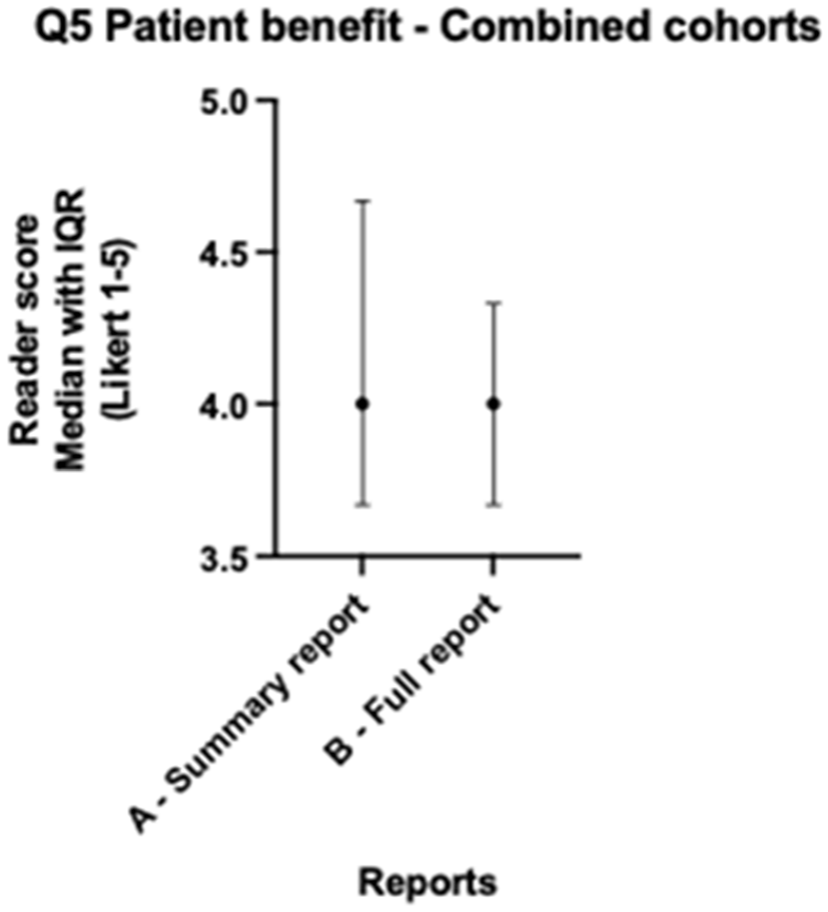
Radiologist colorectal & lung - Patient benefit median scores

**Fig. 5:**
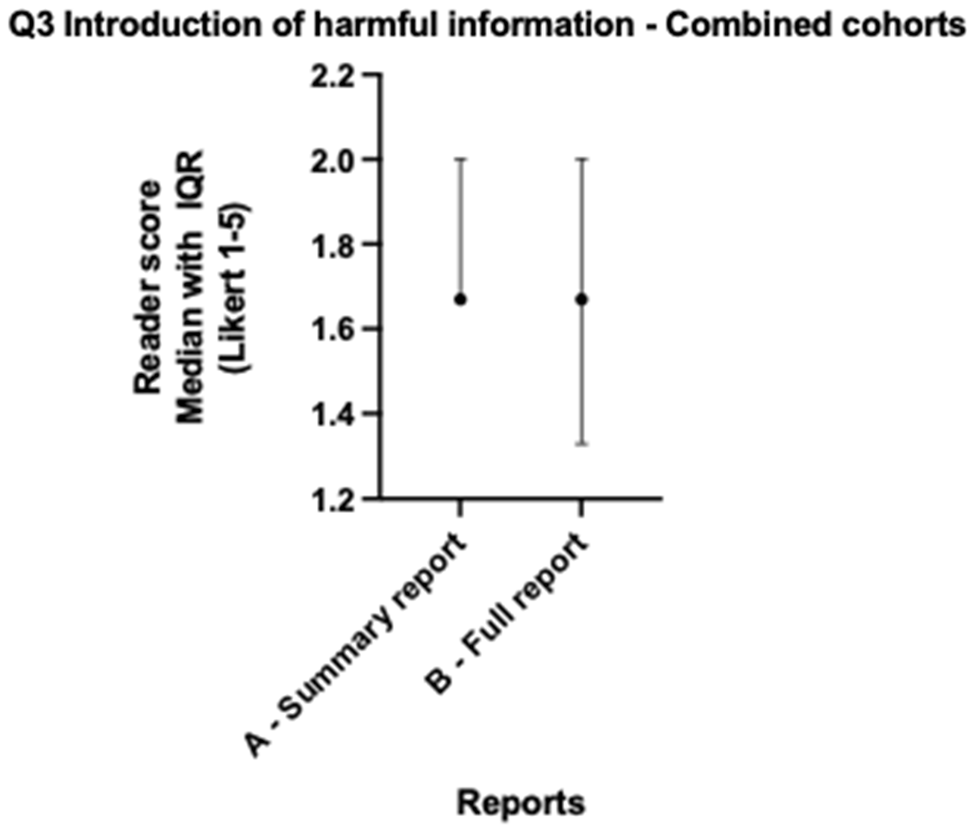
Radiologist colorectal & Lung - Introduction of harmful information median scores

In the lung cancer cohort, Wilcoxon signed-rank tests showed significant differences between version A and version B across most questions (see additional file 3. Table 1 and additional file 4. Figures 1, 2, 3, 4 and 5). Version A was rated higher for factual correctness (*P* = 0.001), inclusion of relevant clinical information (*P* < 0.0001), accessibility/readability for patients (*P* = 0.026), and perceived benefit to patients (*P* < 0.0001). Conversely, version B was considered more likely to include potentially harmful information (*P* = 0.001).

In the colorectal cohort, opposite results are seen across all readers. Version B achieved consistently higher scores for questions 1, 2, 4 and 5 (all *P* < 0.002; see additional file 3. Table 2 and additional file 4. Figures 6, 7, 8, 9 and 10). Version B was also perceived to have less harmful information than version A (*P* = 0.001).

When lung and colorectal cohorts are combined, median scores for all readers across both cohorts for version A and version B showed no statistically significant differences for all five questions (all *p* > 0.057) as per Table 3. Both version A and B scored > 4 (Agree) across factual correctness (Fig. 1), relevant clinical information (Fig. 2), accessibility and readability (Fig. 3), and patient benefit (Fig. 4), and for the introduction of harmful information (Fig. 5), both versions scored ≤2 (Disagree). The differences in both cancer cohorts are cancelled when combined, a possible case of Simpson’s paradox [17], hence the importance of analysing the cohorts separately.

Repeatability was evaluated on a sub-set of six reports by the same three readers. For both cohorts, results show that across all questions, no significant differences were observed between version A and version B (see additional file 3. Table 3 Lung and Table 4 Colorectal).

**Table 3:** Radiologist colorectal & lung - Median scores across readers and Wilcoxon tests

Question	Version A-Summary Median (IQR)	Version A Summary % Scores Q1, Q2, Q4, Q5 > 4 Q3 ≤2	Version B Full-length Median (IQR)	Version B Full-length % Scores Q1, Q2, Q4, Q5 > 4 Q3 ≤2	Median difference (B-A)	Wilcoxon W (B-A)	*P*-value (B-A)
Question 1: Version is factually correct?	4.330 (3.670–4.670)	65%	4.330 (4.000–4.670)	70%	0.000	-2.000	0.995
Question 2: Version includes relevant clinical information?	4.500 (4.000-4.670)	72%	4.670 (4.330–4.670)	87%	0.000	313.000	0.057
Question 3: Version includes harmful information, which might result in physical and/or psychological harm?	1.670 (1.670-2.000)	77%	1.670 (1.330–2.000)	87%	0.000	-134.000	0.386
Question 4: Version is accessible and readable for a patient/lay person?	4.330 (4.000- 4.670)	72%	4.670 (4.330–4.670)	80%	0.000	232.000	0.144
Question 5: Version is beneficial to patients, in addition to the original report?	4.000 (3.670–4.670)	45%	4.000 (3.670–4.330)	30%	-0.330	-393.000	0.127

### PPI review

In the lung cohort, Friedman test showed significant overall differences across the three versions for all three questions (see additional file 3. Table 5 and additional file 4. Figures 11, 12 and 13). Post-hoc Wilcoxon signed-rank tests revealed that version B was rated significantly higher than version A for accessibility (Q1: *P* = 0.047) and being beneficial to patients (Q2: *P* = 0.004), B was the most preferred version for access (Q3: *P* = 0.016).

In the colorectal cohort, Friedman test identified significant differences across the three versions for all questions (all *P* < 0.001) (see additional file 3. Table 6 and additional file 4. Figures 14, 15 and 16). For accessibility and readability (Q1), both versions A and B were rated significantly higher than the original version, although post-hoc Wilcoxon signed-rank tests showed no significant difference between A and B (*P* = 0.57). As to benefit to patients (Q2), A and B scored higher than the original, however they did not differ significantly (*P* = 0.27). For preference of access, B was the most preferred version (Q3: *P* = 0.004).

In all questions, combined colorectal and lung cohorts, the Friedman test showed significant differences between the original version, version A, and version B reports (all *P* < 0.0001), with the original scoring less for accessibility and being beneficial to patients, in comparison with versions A and B as per Table 4.

Pairwise analysis between versions A and B revealed that version B scored significantly higher for accessibility (Q1: *P* = 0.029; see additional file 4. Figure 17) and for being beneficial to patients (Q2: *P* = 0.006; see additional file 4. Figure 18). Version B was the most preferred option (Q3: *P* < 0.0001; see additional file 4. Figure 19).

**Table 4:** PPI Colorectal and Lung - Median scores across readers, Friedman and Wilcoxon tests

Question	Original version Median (IQR)	Original version % Scores Q1 & Q2 > 4 Q3≤1	Version A Summary Median (IQR)	Version A Summary % Scores Q1 & Q2 > 4 Q3≤1	Version B Full-length Median (IQR)	Version B Full-length % Scores Q1 & Q2≥4 Q3≤1	Friedman X2	*P*-value (overall)	Median difference (B-A)	Wilcoxon W (B-A)	*P*-value (B-A)
Q1: Accessibility and readability for a patient/lay person	1.667 (1.333–1.667)	0%	4.333 (3.667–4.333)	60%	4.333 (4.333–4.333)	85%	32.5300	< 0.0001	0.3333	84.0000	0.0286
Q2: Beneficial to patients	3.000 (2.667–3.333)	0%	3.667 (3.333–3.667)	10%	4.000 (3.667–4.000)	10%	30.5000	< 0.0001	0.3333	129.0000	0.0064
Q3 Would like to have access to the version of the report	1.333 (1.333–1.333)	0%	1.333 (1.333–1.333)	0%	1.000 (1.000–1.333)	70%	26.7000	< 0.0001	-0.3333	-136.0000	< 0.0001

### Qualitative data

Open comments from all readers, collected as part of the reader analysis, were coded inductively and summarised as per Table 5, with corresponding quotes to illustrate the respective code. Incorrect facts and statements in versions A and/or B were the most common open comment (*n* = 28), followed by complex terminology (*n* = 18), adding confusion (*n* = 10) as well as missing information (*n* = 10).

**Table 5:** Qualitative analysis from open comments

Code	Description	Lung (Radiologists)		Colorectal (Radiologists)		Lung & Colorectal (PPI)	Total	Quotes radiologists
		Version A	Version B	Version A	Version B			
1 Adds confusion	Difficult interpretation and understanding	*n* = 3	*n* = 3	*n* = 3	*n* = 1	*n* = 0	*n* = 10	(Lung; Version B) “…feel the translation of a thyroid goitre into a ‘lumpy thyroid’ may confuse a patient…”
2 Adds harm	Adds concerns, psychological distress and harm	*n* = 3	*n* = 1	*n* = 0	*n* = 2	*n* = 0	*n* = 6	(Lung; Version A) “I think referring to a haemangioma in the liver as ‘tumour’ might be distressing to the patient and make them think they have another cancer.”
3 Factually incorrect	Incorrect facts and statements added to the outputs	*n* = 4	*n* = 8	*n* = 9	*n* = 7	*n* = 0	*n* = 28	(Colorectal; Version A) “My interpretation of the summary is that there is disease outside the liver which is stable. This is not factually correct. ”
4 Complex terminology	Not simplified enough	*n* = 6	*n* = 3	*n* = 6	*n* = 3	*n* = 0	*n* = 18	(Colorectal; Version A) “still lots of jargon e.g. peritoneal fat, transverse colon, splenic flexure etc”
5 Misleading simplifications	Reduces clinical accuracy	*n* = 0	*n* = 1	*n* = 4	*n* = 3	*n* = 0	*n* = 8	(Colorectal; Version B) “describing the metastases as “lung cancer spots” is potentially misleading. A patient may interpret this to mean that they have lung cancer (rather than metastatic bowel cancer).”
6 Missing information	Outputs are incomplete, important details omitted	*n* = 0	*n* = 0	*n* = 6	*n* = 3	*n* = 1	*n* = 10	(Colorectal; Version A) “Summary does not have an opinion and has omitted the finding of lymphangitis.”
7 Excessive information	Unnecessary details in the report	*n* = 0	*n* = 0	*n* = 1	*n* = 2	*n* = 1	*n* = 4	(Colorectal; Version A) “Personally, I think the summary is too detailed. For example, would a patient need to know that the abdominal organs are normal. If it were me I would just like to know if the disease is better or worse and any important incidental findings conveyed as concisely and clearly as possible.”
								**Quotes PPI**
8 - Personal preferences	Depends on Individual cognitive capability and preference between version A, version B or original report	*n* = 0	*n* = 0	*n* = 0	*n* = 0	*n* = 8	*n* = 8	“…I don’t think one can assume all patients have the same level of health literacy and we should assure that the amount of information a patient receives about their scan result should ideally be tailored to their individual needs and preferences.” “If a patient chooses to see his/her original report, which I would do, it is critical that the patient understands the report. These reports are not written in patient friendly language. The content therefore needs to be explained by his/her consultant, who can explain the findings and consequences of such findings and proposed treatment plan. This can empower patients and allow patients to be more involved in their care.”
9 Discussion with clinical team	Clinical team best placed to discuss with the patient the preferred level of information and content of original report	*n* = 0	*n* = 0	*n* = 0	*n* = 0	*n* = 2	*n* = 2	“If a patient chooses to see his/her original report, which I would do, it is critical that the patient understands the report. These reports are not written in patient friendly language. The content therefore needs to be explained by his/her consultant, who can explain the findings and consequences of such findings and proposed treatment plan. This can empower patients and allow patients to be more involved in their care.”

## Discussion

The increasing implementation of Digital Health Records in the UK and worldwide, is gradually changing how patients access their medical records, including imaging reports. Recently published guidance by the National Health Service England [18] in conjunction with the Society of Radiographers (SoR) and the Royal College of Radiologists (RCR) in March 2025, sets out core principles for effective communication of imaging results, of which “*the imaging report is a medical opinion and so will contain medical phrases and terminology. RCR is working to recommend adjustments to imaging reports in light of greater patient access*,* and the report may include a footnote to this effect*”. The European Society of Radiology (ESR) commitment to value-based radiology, published in 2023 [19] presented the value of structured reporting to patients and HCP and identified that despite the evidence as potential improvers of patient understanding and satisfaction over the past years, structured reporting has not yet embedded itself into clinical practice.

To date, radiology reports have largely remained unchanged, being written by clinicians for clinicians. Patients, however, are increasingly becoming “end receivers and customers”, such as in the United States, with the implementation of the 21st Century Cures Act [20] and in the Netherlands since July 2020^4^. Whilst still a gradual process across the UK, this SE aimed to give an early overview of both healthcare professionals and patient’s views within an oncological setting, which is clinically complex and differs from general radiology reports. This SE assessed the capability of an LLM to generate simplified oncology imaging reports in two report formats. It also aimed to compare how these two formats could impact accuracy, clarity and perceived patient benefit, as well as any perceived harm introduced by the LLM.

Separate analysis per cohort, reveals radiologists in the lung cohort scored version A (summary) more favourably, whereas in the colorectal cohort, version B (full-length) received higher scores. Nonetheless, version A and B separate cohort median scores across correctness, relevant clinical information, accessibility and readability were all > 4 (Agree). An exception was observed in the colorectal cohort, where version A had a median score of 3.670 for patient benefit. Repeatability results for both cohorts showed no significant difference. These differences in radiologist preference per version are supported by the qualitative data. In the colorectal cohort, a higher frequency of recurring codes, including complex terminology and missing information for the summary reports, was observed, suggesting the preference for full-length simplifications.

As for PPI results, the combined quantitative analysis across both cohorts indicates version B as the preferred version. Separate analysis per cohort presents version B more favourably in the lung cohort and in the colorectal cohort there were no differences between versions. The PPI readers all had previous experience and knowledge of imaging research which may have introduced an element of confirmation bias. The qualitative data, however, provided further invaluable insights which are well-illustrated by the following patient quote: “…*My preference for the summary vs full report will therefore depend on what I want to know at any given time and who else was also needing to read the report at that time; even then*,* they may be different i.e. I want to read the full report and my husband the summary report…”*.

These favourable results for the LLM-simplified reports are in line with recent studies conducted to assess how clinicians and wider HCP can support patients and their families in understanding their imaging reports with the use of AI tools [4], with similar results to the accuracy of the outputs [15, 21–23]. New technologies are emerging very quickly to include LLM that can combine images and text to support radiology report interpretation. Assessment of such tools should address not only technical performance but also include patient-reported outcome measures, health economic evaluations, and considerations for the regulatory frameworks, to guarantee that these tools are sustainably, as well as safely, implemented into clinical practice.

Our results do indicate that one size does not fit all, preferences vary and this must be acknowledged when developing new tools. Whilst previously such requirements would be hard to fulfil [24], AI tools could be used to tailor imaging reports according to patient and HCP preferences, whilst maintaining report integrity. Nonetheless, this study does highlight that, in some instances, the LLM generated factually incorrect or potentially harmful information which has also been highlighted by other groups [25]. In the neuroradiology setting, for example, omissions and incorrect emphasis highlight the need for ongoing professional validation and oversight. [26]. Adding validation of LLM generated reports to radiologist’s workload would not be feasible under current extreme workforce pressures. Therefore, in order for these tools to become standard operational practice, it is imperative that radiologists’ time and workflow is not compromised [27, 28] and that LLM in oncology are developed and validated to the point of safe use without the need for substantial manual checking.

The Prompt-development phase was carefully engineered and conducted by radiology experts and data analysts to ensure the two best performing prompts were used only once to generate both versions for the review. Nevertheless, the variability observed during the Prompt-development phase in the outputs generated from different prompts underscores the necessity for ongoing efforts by LLM providers to establish standardisation and best practice guidelines. Such measures are essential to enhance the rigour and reproducibility of LLM research within the healthcare sector, thereby making a meaningful contribution to the advancement of future studies in this field, as highlighted by Patwardhan et al. [29].

In this SE only a single LLM was assessed and the total number of original imaging reports explored was modest, and pertained to two cancer types, colorectal and lung, from a single institution. Nevertheless, a depth of interrogation including multiple detailed assessments and reads was undertaken by both HCP and PPI. Furthermore, both full-length and summary LLM generated outputs were assessed by the two groups. The PPI review also included the original reports, alongside version A and B.

The assessment of LLM in oncology imaging has been underrepresented in the rapidly growing LLM literature, but it is highly relevant and has a specific context. Oncology patients are often faced with large volumes of imaging results, containing technical language, and with implications relating to their survival. Understanding their imaging reports is a crucial element for patient-centred care and shared decision making, in line with the introduction of Evidence-Based Patient Centric Care (EBPCC) in the cancer care pathway [30].

In tandem with new tools, evolving roles for both clinicians and wider healthcare professional community are gaining momentum [31]. Radiology, which was first implemented in the 1940s in response to technological advancements and the need for specialist interpretation, is once again at a pivotal juncture. Today, radiology is being reshaped to reach a broader audience through the adoption of increasingly complex and dynamic technologies.

## Conclusions

Exploring clinician and patient perspectives, this SE suggests that LLM-simplified reports have the potential to improve patient’s access and understanding of imaging reports. Examples of incorrect and potentially harmful information generated by the LLM highlight the need for improvement in the oncology setting, particularly in the absence of radiology workforce capacity for manual checking. Caution should be exercised with regards to patients’ independent use of LLM without HCP support for discussion. While radiologists’ preferences differed between cohorts, with the lung cohort preferring the summary version and the colorectal cohort preferring the full-length version, patients’ overall preference was for the latter. These differences indicate that report outputs will likely need to be tailored to individual patients and HCP preferences.

## Supplementary Information

Below is the link to the electronic supplementary material.

Supplementary Material 1

Supplementary Material 2

Supplementary Material 3

Supplementary Material 4

## Data Availability

Data supporting the findings of this study are available within the paper and its supplementary information files, except for the original anonymised CT reports for colorectal and lung and respective generated version A and B, as part of the multi-reader phase. These can be made available upon reasonable request and permission from the Committee for Clinical Research (CCR) - Royal Marsden NHS Foundation Trust.

## Abbreviations

DHR: Digital Health Records
LLM: Large Language Model(s)
SE: Service Evaluation
PPI: Patient and Public Involvement
HCP: Healthcare professionals
CT: Computed Tomography
RM: The Royal Marsden NHS Foundation Trust
CCR: Committee for Clinical Research
NDOO: National Data Opt-Out
IQR: Interquartile ranges
SD: Standard deviations
W: Wilcoxon signed-rank test statistics
Q(n): Question (number)
SoR: Society of Radiographers
RCR: Royal College of Radiologists
ESR: European Society of Radiology
UK: United Kingdom
EBPCC: Evidence-Based Patient Centric Care
