# Supplementary material for: Improving patient understanding of oncology imaging: radiologist and patient evaluation of summarised versus full-length AI-simplified reports from a tertiary cancer centre
Source: Cancer Imaging. 2026 Apr 13;26:68. doi: 10.1186/s40644-026-01031-x (PMC13188515; doi:10.1186/s40644-026-01031-x)
Supplement: Supplementary file 1 — Supplementary Material 1 [file 40644_2026_1031_MOESM1_ESM.docx]

# Additional file 1. Prompt development steps

| **Step 1**  Initial 30 prompts (A=15; B=15) were edited from published papers (Jeblick et al., 2023; Lyu et al., 2023), iteratively developed using OpenAI 4 GPT-4 , Llama 3 8b and Mistral 7b, as well as manual free text developed iteratively and empirically by the data analysts (SM, SC). Prompts that scored 4 and above by the two reviewers were selected for next step. | | |
| --- | --- | --- |
| **Prompt** | **Prompt used** | **Reports** |
| 1 | " You will be provided with a text. The text is the imaging report of a patient. Your task is to extract the key points in the report accurately in plain language. | A & B |
| 2 | " You will be provided with a text. The text is the imaging report of a patient. Your task is to summarise the text in 3 sentences. | A & B |
| 3 | " You will be provided with a text. The text is the imaging report of a patient. Your task is to summarise the text in 3 paragraphs. | A & B |
| 4 | "You will be provided with a medical imaging report. Your task is to summarize the report into plain language that is easy for the average person to understand. Your response should provide a clear summary of the key findings in the report, using simple language that avoids medical jargon. Please note that your summary should accurately convey the information contained in the original report while making it accessible and understandable to a layperson. You may use analogies or examples to help explain complex concepts, but you should avoid oversimplifying or leaving out important details. | A & B |
| 5 | "You will be provided with a text. The text is the imaging report of a patient. Summarise this radiology report in a single paragraph.  The summary should be in non-medical language and avoid medical jargon. It should be accurate including all information and no new information. | A & B |
| 6 | "Generate a clear and succinct summary of this medical imaging report in professional language. Include important findings, potential diagnoses, and any suggested next steps, framed in a way that can be easily explained to a patient." | A & B |
| 7 | "Provide a brief and complete, patient-friendly summary of the imaging report, highlighting the main results, any abnormalities detected, and the next steps in the patient's care plan. Keep the language professional and clear.” | A & B |
| 8 | "Write a professional yet straightforward summary of the medical imaging report, focusing on the major findings, their relevance to the patient’s condition, and any required next steps or treatments. Avoid excessive technical language while ensuring clarity and accuracy.” | A & B |
| 9 | "You will be provided with a medical report. Your task is to summarize the report using professional language that can be easily understood by the average person. You should not leave any important point out and explain difficult concepts." | A & B |
| 10 | "Summarize the key findings from this medical imaging report for the patient, explaining any significant observations or concerns in a professional and clear manner. Be concise but cover all necessary details regarding diagnosis and treatment recommendations. the summary should be in  the third person and avoid repetition." | A & B |
| 11 | “You will be provided with a text. The text is a medical imaging report. Your task is to summarise the report in non-medical language.” | A & B |
| 12 | "Can you provide a comprehensive summary of the given radiology report? The summary should cover all the key points presented in the original report, while also condensing the information into a concise and easy-to-understand language. The summary should make sense to a non-medical audience. The length of the summary should be appropriate for the length and complexity of the original report, providing a clear and accurate overview without omitting any important information." | A & B |
| 13 | "Could you summarize and translate the provided radiology report into simpler, non-medical language? The summary should capture all the main findings and essential information from the report, making it easy to understand for someone without a medical background. Please ensure that the summary maintains accuracy by including relevant details and examples, while avoiding technical jargon and unnecessary repetition." | A & B |
| 14 | "Please summarize the following radiology report for a non-medical audience. Avoid using medical jargon and instead, explain the findings in simple, everyday language. Focus on what the results mean for the patient and if there are any concerns that need attention. If any recommendations  or next steps are mentioned, please include those as well in a clear, straightforward way." | A & B |
| 15 | "I have a radiology report with complex medical terminology. Please provide a brief, clear summary of the report in simple, non-medical language that a patient with no medical background can understand. Maintain a professional tone and focus on summarizing the key findings and their  implications, avoiding medical jargon. If recommendations or next steps are mentioned, include them in the summary as well." | A & B |
|  | Prompt B - Full Report translation | A & B |
| 16 | "You will be provided with a text. The text is the imaging report of a patient. Your task is to translate the text accurately for a non-medical-audience. " | A & B |
| 17 | "Your role is a that of a medical professional. Your task is to provide an unbiased explanation in common language of the medical report you will be provided. " | A & B |
| 18 | "You will be provided with a text. The text is a medical imaging report. Your task is to translate the report into plain language that is easy for the average person to understand. Your response should provide a clear and concise translation of the key findings in the report, using simple language that avoids medical jargon. Please note that your translation should accurately convey the information contained in the original report while making it accessible and understandable to a layperson. You may use analogies or examples to help explain complex concepts, but you should avoid oversimplifying or leaving out important details." | A & B |
| 19 | "You will be provided with a medical imaging report. Please help translate the report into plain language in the following format: - First paragraph introduces screening description including reason for screening, screening time, protocol, patient background, and comparison date; - Second paragraph talks about specific findings. Please don’t leave out any information about findings. - Third paragraph draws conclusions. - If there are incidental findings, please introduce them in the fourth paragraph Your translation should maintain a professional tone and be clear to a non-medical audience." | A & B |
| 20 | "Could you translate the provided radiology report into simpler, non-medical language? The translation should capture all the main findings and essential information from the report, making it easy to understand for someone without a medical background. Please ensure that the translation maintains accuracy by including relevant details and examples, while avoiding technical jargon and unnecessary repetition. The length of the summary should be appropriate for conveying the content of the original report effectively. " | A & B |
| 21 | "Translate this medical imaging report into patient-friendly language. Ensure that all key results and their implications are clearly explained, without using confusing medical jargon. Focus on helping the patient understand what the results mean for their health in a professional and approachable tone." | A & B |
| 22 | "Translate the following medical imaging report in a way that is easy for a non-medical audience to understand. Break down complex medical terms into simple explanations, covering all critical findings. Keep the tone professional and neutral." | A & B |
| 23 | "Provide a plain-language summary of this medical imaging report for a patient. Explain the main findings in clear, everyday language, focusing on what the results mean for their health. Ensure that the tone is professional and informative but not overly technical. " | A & B |
| 24 | "Translate the following medical imaging report into simple and concise language that a patient can easily understand. Summarize the key findings and explain what they mean in non-technical terms, while maintaining a professional tone. Avoid using medical jargon but ensure that the information remains accurate. " | A & B |
| 25 | "Reword this medical imaging report so that a patient can fully understand their results without needing medical knowledge. Explain the results in plain language, focus on what is most relevant without leaving any important information out. You should use a professional, impersonal tone." | A & B |
| 26 | "Can you provide a layman's interpretation of the provided radiology report? The interpretation should simplify the medical terminology and concepts into everyday language that is accessible to a general audience. Please ensure that the interpretation captures all the main findings and significant details from the report, while avoiding complex terminology and unnecessary technical explanations. The goal is to make the content of the report understandable to those without medical knowledge." | A & B |
| 27 | "I have a radiology report that contains complex medical terminology. Please rewrite the following report in simple, non-medical language so that it can be easily understood by a patient with no medical background. Keep the description accurate but avoid jargon. Make sure to explain any  findings in a clear, patient-friendly manner." | A & B |
| 28 | "I have a radiology report containing medical terminology. Please rewrite the report in clear, simple language that a patient with no medical background can easily understand. Maintain a professional tone and ensure that the description remains accurate without using medical jargon.  Provide a clear explanation of the findings and their implications." | A & B |
| 29 | "I have a radiology report that contains complex medical terminology. Please translate the entire report into clear, simple language without using medical jargon, so that a patient with no medical background can fully understand it. Maintain a professional tone, ensuring the explanation is accurate, while focusing on making the content accessible and understandable. Include explanations of any findings, their significance, and any recommended actions or next steps." | A & B |
| 30 | "I need to make a radiology report understandable for someone without a medical background. Please convert the entire report into plain, everyday language, avoiding any medical jargon. Ensure the translation is clear and professional, accurately reflecting the findings and their  significance. Include explanations of any results and what they mean, as well as any suggested follow-up actions or recommendations." | A & B |

| **Step 2**  Four prompts reviewed, and two prompts selected 6 (A summary) and Prompt 28 (B full simplification) based on scores and consensus between reviewers. | | |
| --- | --- | --- |
| **Prompt** | **Prompt used** | **Reports** |
| 2 | You will be provided with a text. The text is the imaging report of a patient. Your task is to summarise the text in 3 sentences. | C |
| 6 | "Generate a clear and succinct summary of this medical imaging report in professional language. Include important findings, potential diagnoses, and any suggested next steps, framed in a way that can be easily explained to a patient." | C |
| 16 | "You will be provided with a text. The text is the imaging report of a patient. Your task is to translate the text accurately for a non-medical audience. " | C |
| 28 | "I have a radiology report containing medical terminology. Please rewrite the report in clear, simple language that a patient with no medical background can easily understand. Maintain a professional tone and ensure that the description remains accurate without using medical jargon.  Provide a clear explanation of the findings and their implications." | C |

| **Step 3**  Five variations of prompt 6 and prompt 28 were developed by editing the prompt wording manually and with suggestions from ChatGPT for certain cases, to align with the criteria as per table 1, performed by SM and AR. 20 outputs were scored by four reviewers. Selected prompt for A (6.1) and prompt for B (28.5). | | |
| --- | --- | --- |
| **Prompt  (Prompt 6 and 28 each had 5 variations)** | **Prompt used** | **Reports** |
| 6.1 | "Generate a clear and succinct summary of this medical imaging report in professional language. Include important findings, potential diagnoses, and any suggested next steps, framed in a way that can be easily explained to a patient." | D & E |
| 6.2 | "Generate a clear and succinct summary of this medical imaging report in professional language. Include important findings, potential diagnoses, and any suggested next steps, framed in a way that can be easily explained to a patient. Your summary should avoid repetition and be in  the third person. Your summary should be two paragraphs or less." | D & E |
| 6.3 | "Generate a concise, third-person summary of this medical imaging report using professional language. Highlight key findings, potential diagnoses, and recommended next steps, ensuring the information is presented clearly and in a way that can be easily explained to a patient. The summary should be no more than two paragraphs and free of repetition." | D & E |
| 6.4 | "Summarize this medical imaging report in clear, professional language, focusing on key findings, potential diagnoses, and recommended next steps. The summary should be succinct, written in the third person, avoid repetition, and be no longer than two paragraphs. Ensure it is understandable to a patient while maintaining a professional tone." | D & E |
| 6.5 | "Provide a concise, third-person summary of this medical imaging report, emphasizing the most important findings, potential diagnoses, and suggested next steps. The summary should be no longer than two paragraphs, avoid repetition, and use professional yet patient-friendly language that is easy to understand." | D & E |
| 28.1 | "I have a radiology report containing medical terminology. Please rewrite the report in clear, simple language that a patient with no medical background can easily understand. Maintain a professional tone and ensure that the description remains accurate without using medical jargon.  Provide a clear explanation of the findings and their implications." | D & E |
| 28.2 | "I have a radiology report containing medical terminology. Please rewrite the report in clear, simple language that a patient with no medical background can easily understand. Maintain a professional tone and ensure that the description remains accurate without using medical jargon.  Your translation should be in the third person, avoid repetition, and keep the structure of the original report. Keeping to the structure of the original report means using the same headings that were used in the report before the start of the section.  Provide a clear explanation of the findings and their implications." | D & E |
| 28.3 | "Translate this radiology report in clear, everyday language ensuring the information is easy to understand and does not use difficult medical jargon. Maintain a professional tone and provide an accurate explanation of the findings and their implications. The translation should be in the third person, avoid repetition, and clearly convey the results to a patient with no medical background. The translation should keep to the structure of the original report and use the same section headings. " | D & E |
| 28.4 | "Translate this radiology report in clear, simple language that a patient with no medical background can easily understand. Keep to the structure of the original report by using the same section labels and provide an accurate third-person explanation of the findings and their meaning.  Avoid ordered lists and bullet points unless they were used in the original text. Avoid medical jargon, repetition, and ensure the tone remains professional." | D & E |
| 28.5 | "Rewrite this radiology report in clear, professional, simple language that a patient with no medical background can easily understand.  Keep the structure of the original report and where applicable use the same headings like for example: 'Background', 'Clinical History', Findings', 'Opinion', 'Conclusion', and so on. Provide an accurate, third-person explanation of the findings and their meaning. Avoid medical jargon, repetition, and ensure the tone remains professional. Do not address the patient directly." | D & E |

| **Step 4**  Few-shot prompting was used, based on 3 new report examples with varying levels of complexity (Appendix A2_ Reports used for prompt development). A-summaries and B-full simplifications using prompts from step 3, and hyperparameter default settings, were manually edited and revised by the 4 reviewers prior to being used as examples. One output had to be regenerated due to a limit in the output tokens, small differences were noted, even though temperature was set to zero, as even with temperature as zero, the output was not deterministic. Comparison of the baseline zero-shot prompts to its three-shot variations as performed and the best performing variation for A (6.1 three shot) and B (28.5 zero shot) were selected. | | |
| --- | --- | --- |
| **Prompt # (6.1 and 28.5)** | **Prompt used** | **Reports** |
| 6.1 (zero shot) | "Generate a clear and succinct summary of this medical imaging report in professional language. Include important findings, potential diagnoses, and any suggested next steps, framed in a way that can be easily explained to a patient." | F & G |
| 6.1 (three shot) | "Generate a clear and succinct summary of this medical imaging report in professional language. Include important findings, potential diagnoses, and any suggested next steps, framed in a way that can be easily explained to a patient. Here are three examples of how the summary should be generated: Example 1 Text : CT Thorax abdomen and pelvis with contrast Clinical information: Metastatic colorectal cancer with left hemicolectomy. Liver and lung metastases. Rising CEA. Findings: Comparison made with a previous CT scan. The bilateral pulmonary metastases are stable in size. No new lung lesions are identified. There are no pleural effusions. There are no enlarged thoracic lymph nodes. There is marked fatty infiltration of the liver making it difficult to reliably compare the burden of metastatic disease, however there has been no appreciable change in the size or number of deposits. There is no biliary duct dilatation. On the remaining solid abdominal viscera are unremarkable. The bowel is unobstructed, and the right-sided stoma is unremarkable. The bilateral ovarian metastases are also stable in size. There are no enlarged abdominal or pelvic lymph nodes. Within the limits of CT there is no metastatic bone disease identified Opinion: Stable appearance of the lung, liver and ovarian disease on CT. Should the reporting radiologist or nuclear medicine physician not verify this report, the report has only been checked for spelling and grammar.  Example 1 Summary: Colorectal cancer that has spread to liver, lungs and ovaries. This scan was compared with the previous one and the cancer in the lungs and ovaries has not grown or spread. The liver has a lot of fat, making it hard to compare with the previous scan but it does not seem  to have changed. No blockage in the intestine and the stoma (part of the intestine that was rerouted to an opening in the abdomen) looks normal. Lymph nodes and bones look normal.  Example 2 Text : CT Thorax abdomen and pelvis with contrast Clinical information: Metastatic signet-ring adenocarcinoma of the appendix with resected Krukenburg tumours and right hemicolectomy. Findings: Comparison is made to previous CT. There is no metastatic disease in the thorax. There is diffuse fatty infiltration of the liver, but no focal lesions are seen. The remaining solid abdominal viscera are unremarkable in their appearance. The bowel is unobstructed.  Previous BSO is noted.  Scans show encysted peritoneal fluid appearances are highly likely to represent progressive disease. The right pelvic sidewall lymph node has increased in size from x mm to x mm. Within the limits of CT no metastatic bone disease is seen. Opinion: Progressive enlargement of the bilateral pelvic cystic fluid and right pelvic sidewall lymph node: suggestive of disease progression Should the reporting radiologist or nuclear medicine physician not verify this report, the report has only been checked for spelling and grammar.  Example 2 Summary: Specific cancer of the appendix, called ""signet ring adenocarcinoma"", which has spread to other parts of the body with previous  operation to remove some of these cancers. This scan shows a normal chest and liver, although the latter shows an abnormal amount of fat.  The organs in the abdomen look normal and no sign of blockage in the intestine. There is fluid in the lining of the abdomen, which is likely a sign that the cancer in progressing. A lymph node (part of the immune system) in the pelvic area has grown in size, which could also indicate  that the disease is progressing. There are no signs of cancer in the bones.  Example 3 Text : CT Thorax abdomen and pelvis with contrast Clinical information: Stage IV non-small cell lung cancer. Follow-up. Previous cyberknife right xth rib. Findings: Comparison is made with previous study. Expansile lesion of the right lateral xth rib remains stable since previous study.  There is new postradiation changes in the adjacent lung parenchyma and there is a new small loculated right pleural effusion and pleural thickening.  There few small nodules in the right lung larger one in the right base measuring xx.xmm previously marginally smaller x.xmm. Very small <xmm nodules in the LUL and LLL remain stable since previous study. Limited soft tissue anterior to the innominate vein in the mediastinum remains stable with no interval change. No new thoracic lymphadenopathy noted. Below the diaphragm no focal liver lesions seen. Small 15 mm cystic lesion in the right adrenal (adenoma) remains stable since previous studies. The spleen, pancreas, both kidneys and left adrenal appear normal.  There is some thickening and irregularity along the course of the left ovarian vein which is non-specific and stable since previous studies and will require further characterisation with MRI. There is a bulky fibroid uterus with an area of fat adjacent to it which could represent an ovarian dermoid or degenerated fibroid. No definite evidence of pelvic lymphadenopathy. No abnormal retroperitoneal or mesenteric lymphadenopathy. Bony review remains stable since previous study. Opinion: Stable appearances of right xth rib lesion. Postradiation changes in the right lung and a new pleural effusion.   Low volume nodularity in the right lung slightly more prominent in the current study and a little more stable on the left lung. Appearances of the lungs suspicious for low volume slow pace progression. Non-specific thickening along the course of the left ovarian vein which could be benign. An MRI could be helpful to characterise further. Should the reporting radiologist or nuclear medicine physician not verify this report, the report has only been checked for spelling and grammar.  Example 3 Summary: Advanced stage lung cancer. Compared to the previous scan, this scan shows some change in the tissue next to xth rib on the  right side, likely due to the radiation treatment. There is also a new small fluid collection and thickening of the lining of the lung on the right  side. The bigger nodule in the right lung appears bigger than before, whereas in the left lung the small nodules are stable. The organs in the abdomen look stable, an MRI could clarify the thickening along the left ovarian vein. The uterus is enlarged due to fibroids, and the area of fat next to it  could be benign ovarian growth or a degenerated fibroid. Lymph nodes in the pelvis appear normal. The bones appear stable. | F & G |
| 28.5 (zero shot) | "Rewrite this radiology report in clear, professional, simple language that a patient with no medical background can easily understand.  Keep the structure of the original report and where applicable use the same headings like for example: 'Background', 'Clinical History', Findings', 'Opinion', 'Conclusion', and so on. Provide an accurate, third-person explanation of the findings and their meaning. Avoid medical jargon, repetition, and ensure the tone remains professional. Do not address the patient directly. | F & G |
| 28.5 (few shot) | "Rewrite this radiology report in clear, professional, simple language that a patient with no medical background can easily understand.  Keep the structure of the original report and where applicable use the same headings like for example: 'Background', 'Clinical History', Findings', 'Opinion', 'Conclusion', and so on. Provide an accurate, third-person explanation of the findings and their meaning. Avoid medical jargon, repetition, and ensure the tone remains professional. Do not address the patient directly. Here are three examples of how the translation should be generated:  Example 1 Text : CT Thorax abdomen and pelvis with contrast Clinical information: Metastatic colorectal cancer with left hemicolectomy. Liver and lung metastases. Rising CEA. Findings: Comparison made with a previous CT scan. The bilateral pulmonary metastases are stable in size. No new lung lesions are identified. There are no pleural effusions. There are no enlarged thoracic lymph nodes. There is marked fatty infiltration of the liver making it difficult to reliably compare the burden of metastatic disease, however there is been no appreciable change in the size or number of deposits. There is no biliary duct dilatation. On the remaining solid abdominal viscera are unremarkable. The bowel is unobstructed, and the right-sided stoma is unremarkable. The bilateral ovarian metastases are also is stable in size. There are no enlarged abdominal or pelvic lymph nodes. Within the limits of CT there is no metastatic bone disease identified Opinion: Stable appearance of the lung, liver and ovarian disease on CT. Should the reporting radiologist or nuclear medicine physician not verify this report, the report has only been checked for spelling and grammar.  Example 1 Simplification: Clinical Information: Advanced colorectal cancer that has spread to the liver and lungs; this scan was compared to a previous one to check for any changes. Findings: Lungs: The cancer lesions in the lungs have not grown or multiplied, and no new cancer lesions were found. There is no fluid build-up around the lungs, and the lymph nodes in the chest area are not swollen. Liver: The fat in the liver, which makes it hard to compare the cancer lesions with the previous scan, however, it seems like there hasn't been any significant change in the size or number of these cancer lesions. The biliary ducts (tubes that carry bile, a digestive fluid in the liver) are not swollen. Abdomen and Pelvis: other organs in the abdomen appear normal. The intestines are not blocked, and the stoma (part of the intestine that was rerouted to an opening in the abdomen) looks normal. The cancer lesions in the ovaries have not grown or multiplied, and the lymph nodes (gland of the immune system) in the abdomen and pelvis are not swollen. No signs of cancer in the bones. Opinion: The CT scan shows that the cancer in the lungs, liver, and ovaries has not worsened. The report has been checked for spelling and grammar, but it should be reviewed by a radiologist or nuclear medicine physician to confirm these findings.  Example 2 Text : CT Thorax abdomen and pelvis with contrast Clinical information: Metastatic signet-ring adenocarcinoma of the appendix with resected Krukenburg tumours and right hemicolectomy. Findings: Comparison is made to previous CT. There is no metastatic disease in the thorax. There is diffuse fatty infiltration of the liver, but no focal lesions are seen. The remaining solid abdominal viscera are unremarkable in their appearance. The bowel is unobstructed.  Previous BSO is noted.  Scans show encysted peritoneal fluid appearances are highly likely to represent progressive disease. The right pelvic sidewall lymph node has increased in size from x mm to x mm. Within the limits of CT no metastatic bone disease is seen. Opinion: Progressive enlargement of the bilateral pelvic cystic fluid and right pelvic sidewall lymph node: suggestive of disease progression Should the reporting radiologist or nuclear medicine physician not verify this report, the report has only been checked for spelling and grammar.  Example 2 Simplification: Clinical information: History of a rare type of cancer that started in the appendix (signet ring adenocarcinoma) and spread to other part of the body. Previous surgery to remove tumours (called Krukenburg tumours) and hemicolectomy (removal of part of the right side of the colon).  Findings: Scan results were compared to previous ones. The chest area doesn't show any signs of cancer spread. The liver shows signs of fat build-up, but no specific areas of concern are seen. The other solid organs in the abdomen look normal. The intestines are not blocked. The patient has had surgery in the past to remove both ovaries and the fallopian tubes.  The scan shows fluid trapped in the lining of the abdomen, which is likely a sign that the cancer is progressing. A lymph node (gland of the immune system) on the right side of the pelvis has grown from x mm to x mm.  There are no signs of cancer spread to the bones within the limits of what the CT scan can detect.   Opinion: the increase in fluid in the lining of the abdomen and the growth of the lymph node on the right side of the pelvis suggest that the cancer may be progressing. This report has been checked for spelling and grammar, but it should be confirmed by the radiologist or nuclear medicine physician who is interpreting the scan.  Example 3 Text : CT Thorax abdomen and pelvis with contrast Clinical information: Stage IV non-small cell lung cancer. Follow-up. Previous cyberknife right xth rib. Findings: Comparison is made with previous study. Expansile lesion of the right lateral xth rib remains stable since previous study.  There is new postradiation changes in the adjacent lung parenchyma and there is a new small loculated right pleural effusion and pleural thickening.  There are few small nodules in the right lung larger one in the right base measuring xx.xmm previously marginally smaller x.xmm. Very small <5mm nodules in the LUL and LLL remain stable since previous study. Limited soft tissue anterior to the innominate vein in the mediastinum remains stable with no interval change. No new thoracic lymphadenopathy noted. Below the diaphragm no focal liver lesions seen. Small xx mm cystic lesion in the right adrenal (adenoma) remains stable since previous studies. The spleen, pancreas, both kidneys and left adrenal appear normal.  There is some thickening and irregularity along the course of the left ovarian vein which is non-specific and stable since previous studies and will require further characterisation with MRI. There is a bulky fibroid uterus with an area of fat adjacent to it which could represent an ovarian dermoid or degenerated fibroid. No definite evidence of pelvic lymphadenopathy. No abnormal retroperitoneal or mesenteric lymphadenopathy.  Bony review remains stable since previous study. Opinion: Stable appearances of right xth rib lesion. Postradiation changes in the right lung and a new pleural effusion.   Low volume nodularity in the right lung slightly more prominent in the current study and a little more stable on the left lung. Appearances of the lungs suspicious for low volume slow pace progression. Non-specific thickening along the course of the left ovarian vein which could be benign. An MRI could be helpful to characterise further. Should the reporting radiologist or nuclear medicine physician not verify this report, the report has only been checked for spelling and grammar.  Example 3 Simplification:  Clinical information: Advanced lung cancer with previous Cyberknife (type of radiation therapy) on the right xth rib Findings:  Chest Area: The abnormal lesion on the right xth rib hasn't changed since the last scan. There are new changes in the lung tissue next to this rib due to the radiation therapy. There is also a new small pocket of fluid and thickening of the lining of the lung on the right side. There are a few small lesions in the right lung, with the largest one slightly bigger (xx.xmm) than before (xmm). The very small lesions in the lower parts of the left lung haven't changed since the last scan. There is no new swelling of the lymph nodes in the chest area. Abdomen: There are no abnormal lesions in the liver. A small, stable cyst in the right adrenal gland (a small gland located above the kidney) is unchanged. The spleen, pancreas, both kidneys, and left adrenal gland look normal. There is some thickening and irregularity in the left ovarian vein (a vein that drains blood from the ovary), which is unchanged but needs further investigation with an MRI.  Pelvis: The uterus has a large fibroid (a non-cancerous growth), and there is an area of fat next to it which could be an ovarian dermoid (a type of benign tumor) or a degenerated fibroid. There is no evidence of swollen lymph nodes in the pelvic area. Bones: The bones look the same as in the previous study.  Opinion: The lesion on the right xth rib is stable. There are changes in the right lung due to radiation therapy and a new pocket of fluid. The small lesions in the right lung are slightly more noticeable, and those in the left lung are stable. The changes in the lungs suggest slow progression of the disease. The thickening of the left ovarian vein could be harmless, but an MRI could provide more information. | F & G |

| **Step 5**  As the model is not deterministic, different runs using the same input report and prompt lead to slightly different outputs, this step was used to confirm that multiple outputs generated using the same prompt and report had comparable scores. Hyperparameters play an important role in the model's output in terms of creativity, repetition, and randomness and tuning them can adjust the output of a model and is therefore an important step to reach a certain target output. In this case the outputs already score >4, therefore focusing on an exploration of hyperparameters, whilst interesting, was not deemed to necessarily improve the outcome. | | |
| --- | --- | --- |
| **Prompt** | **Prompt used** | **Reports** |
| 6.1 (three shot) | "Generate a clear and succinct summary of this medical imaging report in professional language. Include important findings, potential diagnoses, and any suggested next steps, framed in a way that can be easily explained to a patient.  Here are three examples of how the summary should be generated:   Example 1: Example 1 Text : CT Thorax abdomen and pelvis with contrast Clinical information: Metastatic colorectal cancer with left hemicolectomy. Liver and lung metastases. Rising CEA. Findings: Comparison made with a previous CT scan. The bilateral pulmonary metastases are stable in size. No new lung lesions are identified. There are no pleural effusions. There are no enlarged thoracic lymph nodes. There is marked fatty infiltration of the liver making it difficult to reliably compare the burden of metastatic disease, however there is been no appreciable change in the size or number of deposits. There is no biliary duct dilatation. On the remaining solid abdominal viscera are unremarkable. The bowel is unobstructed, and the right-sided stoma is unremarkable. The bilateral ovarian metastases are also is stable in size. There are no enlarged abdominal or pelvic lymph nodes. Within the limits of CT there is no metastatic bone disease identified Opinion: Stable appearance of the lung, liver and ovarian disease on CT. Should the reporting radiologist or nuclear medicine physician not verify this report, the report has only been checked for spelling and grammar.  Example 1 Summary: Colorectal cancer that has spread to liver, lungs and ovaries. This scan was compared with the previous one and the cancer  in the lungs and ovaries has not grown or spread. The liver has a lot of fat, making it hard to compare with the previous scan but it does not seem  to have changed. No blockage in the intestine and the stoma (part of the intestine that was rerouted to an opening in the abdomen) looks normal.  Lymph nodes and bones look normal.  Example 2 Text : CT Thorax abdomen and pelvis with contrast Clinical information: Metastatic signet-ring adenocarcinoma of the appendix with resected Krukenburg tumours and right hemicolectomy. Findings: Comparison is made to previous CT. There is no metastatic disease in the thorax. There is diffuse fatty infiltration of the liver, but no focal lesions are seen. The remaining solid abdominal viscera are unremarkable in their appearance. The bowel is unobstructed.  Previous BSO is noted.  Scans show encysted peritoneal fluid appearances are highly likely to represent progressive disease. The right pelvic sidewall lymph node has increased in size from x mm to x mm. Within the limits of CT no metastatic bone disease is seen. Opinion: Progressive enlargement of the bilateral pelvic cystic fluid and right pelvic sidewall lymph node: suggestive of disease progression Should the reporting radiologist or nuclear medicine physician not verify this report, the report has only been checked for spelling and grammar.  Example 2 Summary: Specific cancer of the appendix, called ""signet ring adenocarcinoma"", which has spread to other parts of the body with previous  operation to remove some of these cancers. This scan shows a normal chest and liver, although the latter shows an abnormal amount of fat.  The organs in the abdomen look normal and no sign of blockage in the intestine. There is fluid in the lining of the abdomen, which is likely a  sign that the cancer in progressing. A lymph node (part of the immune system) in the pelvic area has grown in size, which could also indicate that the disease is progressing. There are no signs of cancer in the bones.  Example 3 Text : CT Thorax abdomen and pelvis with contrast Clinical information: Stage IV non-small cell lung cancer. Follow-up. Previous cyberknife right xth rib. Findings: Comparison is made with previous study. Expansile lesion of the right lateral xth rib remains stable since previous study.  There is new postradiation changes in the adjacent lung parenchyma and there is a new small loculated right pleural effusion and pleural thickening.  There are few small nodules in the right lung larger one in the right base measuring xx.xmm previously marginally smaller x.xmm. Very small <xmm nodules in the LUL and LLL remain stable since previous study. Limited soft tissue anterior to the innominate vein in the mediastinum remains stable with no interval change. No new thoracic lymphadenopathy noted. Below the diaphragm no focal liver lesions seen. Small xx mm cystic lesion in the right adrenal (adenoma) remains stable since previous studies. The spleen, pancreas, both kidneys and left adrenal appear normal.  There is some thickening and irregularity along the course of the left ovarian vein which is non-specific and stable since previous studies and will require further characterisation with MRI. There is a bulky fibroid uterus with an area of fat adjacent to it which could represent an ovarian dermoid or degenerated fibroid. No definite evidence of pelvic lymphadenopathy. No abnormal retroperitoneal or mesenteric lymphadenopathy.  Bony review remains stable since previous study. Opinion: Stable appearances of right xth rib lesion. Postradiation changes in the right lung and a new pleural effusion.   Low volume nodularity in the right lung slightly more prominent in the current study and a little more stable on the left lung. Appearances of the lungs suspicious for low volume slow pace progression. Non-specific thickening along the course of the left ovarian vein which could be benign. An MRI could be helpful to characterise further. Should the reporting radiologist or nuclear medicine physician not verify this report, the report has only been checked for spelling and grammar.  Example 3 Summary: Advanced stage lung cancer. Compared to the previous scan, this scan shows some change in the tissue next to xth rib on the right side, likely due to the radiation treatment. There is also a new small fluid collection and thickening of the lining of the lung on the right  side. The bigger nodule in the right lung appears bigger than before, whereas in the left lung the small nodules are stable. The organs in the abdomen  look stable, an MRI could clarify the thickening along the left ovarian vein. The uterus is enlarged due to fibroids, and the area of fat next to it  could be benign ovarian growth or a degenerated fibroid. Lymph nodes in the pelvis appear normal. The bones appear stable. | H & I |
| 28.5 (zero shot) | "Rewrite this radiology report in clear, professional, simple language that a patient with no medical background can easily understand.  Keep the structure of the original report and where applicable use the same headings like for example: 'Background', 'Clinical History', Findings', 'Opinion', 'Conclusion', and so on. Provide an accurate, third-person explanation of the findings and their meaning. Avoid medical jargon, repetition, and ensure the tone remains professional. Do not address the patient directly." | H & I |
