# Supplementary material for: Improving patient understanding of oncology imaging: radiologist and patient evaluation of summarised versus full-length AI-simplified reports from a tertiary cancer centre
Source: Cancer Imaging. 2026 Apr 13;26:68. doi: 10.1186/s40644-026-01031-x (PMC13188515; doi:10.1186/s40644-026-01031-x)
Supplement: Supplementary file 2 — Supplementary Material 2 [file 40644_2026_1031_MOESM2_ESM.docx]

Additional file 2. Example of Original, Version A and B for colorectal and lung cohorts

Please note for publication purposes, the exact measurements have been removed, however these were part of the report content for prompt development.

| **Cohort** | **Original Report** | **Version A (summary)** | **Version B (full-length)** |
| --- | --- | --- | --- |
| **Colorectal** | CT Thorax abdomen and pelvis with contrast  Clinical information: Metastatic adenocarcinoma of the colon. To assess pace of disease (low volume pulmonary metastases).  Findings: Comparison is made to previous CT dated. There is severe centrilobular and paraseptal emphysematous change with subpleural fibrotic change. There is granulomatous formation with calcification in the left lung apex; soft tissue at this site is unchanged. The deposit in the left lower lobe abutting the oblique fissure has increased from xx mm to xx mm. A nodule along the right horizontal fissure measures xx mm. Subcentimetre mediastinal and right hilar lymph nodes are stable. The solid abdominal viscera are unremarkable in their appearance. The midline anterior abdominal wall hernia is noted. No GI or renal obstruction. No enlarged abdominal or pelvic lymph nodes.  No metastatic bone disease can be seen on CT.  Opinion: There has been further progression of the low volume pulmonary metastatic disease.  Should the reporting radiologist or nuclear medicine physician not verify this report, the report has only been checked for spelling and grammar. | Summary: The patient has colon cancer that has spread to the lungs. Compared to the previous scan, there is severe damage to the lungs due to emphysema and fibrosis. There is also a granuloma (small area of inflammation) with calcification in the left lung, which is unchanged. The cancer in the left lower lung has grown from xx mm to xx mm. A nodule in the right lung measures xx mm. The lymph nodes in the chest are stable. The organs in the abdomen appear normal, and there is a hernia in the anterior abdominal wall. There is no blockage in the gastrointestinal or renal systems, and the lymph nodes in the abdomen and pelvis are normal. There is no sign of cancer in the bones. The cancer in the lungs appears to be progressing. | CT scan of Chest, Abdomen, and Pelvis with Contrast  Background: This patient has a type of colon cancer that has spread to other parts of the body. The purpose of this scan is to check how fast the disease is progressing, particularly in the lungs where there are small amounts of cancer.  Findings: This report is compared to the previous CT scan. There are severe changes in the lungs, specifically in the small air sacs and the walls between them. There are also changes in the outer layer of the lungs. There is a formation with calcification (hardening due to calcium deposits) in the top of the left lung; the soft tissue in this area has not changed. The cancer deposit in the lower left lung has grown from xx mm to xx mm. There is a small growth along the right lung measuring xx mm. Small lymph nodes in the chest and right lung area are stable. The solid organs in the abdomen look normal. A hernia (bulging of an organ or tissue) in the middle front of the abdominal wall is noted. There are no blockages in the gastrointestinal or kidney areas. There are no enlarged lymph nodes in the abdomen or pelvis. No cancer spread to the bones can be seen on the CT scan.  Conclusion: The small amounts of cancer in the lungs have progressed further.  If the radiologist or nuclear medicine doctor who is reporting this does not verify this report, it has only been checked for spelling and grammar. |
|  | | | |
| **Lung** | CT Thorax abdomen and pelvis with contrast Clinical information: ? Progressive adenocarcinoma of lung. Findings: Scans through the trunk following IV contrast are compared with a previous study. Bullous change at the right lung base persists and there is no new pulmonary mass lesion, mediastinal adenopathy or free pleural fluid. Below the diaphragm the very large area of metastatic disease seen superiorly in the right hepatic lobe on the previous study has markedly regressed currently measuring xx x xx cm compared to xx x xx cm previously (compare current image xx with previous image xx). This process involves the posterior capsule of the liver and appears solitary. There is no further hepatic mass lesion or biliary obstruction and the gallbladder, pancreas and spleen remain normal. There is no renal mass lesion or hydronephrosis and no retroperitoneal adenopathy or adrenal lesion. The stomach, small and large bowel are grossly normal, and the pelvic viscera are unremarkable. Review on bony windows shows sclerotic and lytic disease in the lumbar and dorsal spines is stable. There is no new metastatic involvement or cord compression. Opinion: Significant response to therapy in the liver. No change in bony metastatic involvement. No new foci of disease activity seen throughout. Should the reporting radiologist or nuclear medicine physician not verify this report, the report has only been checked for spelling and grammar. | Summary: The patient has adenocarcinoma of the lung, a type of cancer, which may be progressing. The scan shows no new growths in the lungs, and the large area of cancer that had spread to the liver has significantly reduced in size, likely due to successful therapy. The other organs in the abdomen and pelvis appear normal. There are changes in the lumbar and dorsal spines due to the cancer, but these have not worsened. There are no new areas of cancer detected in this scan. | CT scan of Chest, Abdomen, and Pelvis with Contrast  Background: Possible advanced lung cancer  Findings: The scan of the chest, abdomen, and pelvis was done after injecting a contrast dye. This scan was compared with a previous one.  There is a persistent abnormality in the lower part of the right lung, but no new lung masses, swollen lymph nodes in the chest, or fluid around the lungs were found. Below the diaphragm, in the upper part of the right lobe of the liver, the large area of cancer spread seen in the previous scan has significantly reduced in size. It now measures xx x xx cm, compared to xx x xx cm previously. This area is located at the back of the liver and appears to be the only one. There are no other liver masses, blockages in the bile ducts, and the gallbladder, pancreas, and spleen look normal. There are no masses in the kidneys, no swelling, no swollen lymph nodes behind the abdomen, or adrenal gland masses.  The stomach, small and large intestines look normal, and the organs in the pelvis are unremarkable.  A review of the bones shows stable disease in the lower and middle back. There is no new cancer spread or pressure on the spinal cord.  Opinion: There is a significant improvement in the liver due to treatment. There is no change in the cancer spread to the bones. No new areas of disease activity were seen throughout.  Please note, this report has only been checked for spelling and grammar. It should be verified by the reporting radiologist or nuclear medicine physician |
