# Supplementary material for: Improving patient understanding of oncology imaging: radiologist and patient evaluation of summarised versus full-length AI-simplified reports from a tertiary cancer centre
Source: Cancer Imaging. 2026 Apr 13;26:68. doi: 10.1186/s40644-026-01031-x (PMC13188515; doi:10.1186/s40644-026-01031-x)
Supplement: Supplementary file 3 — Supplementary Material 3 [file 40644_2026_1031_MOESM3_ESM.docx]

# Additional file 3. Tables

| **Question** | **Version A-Summary Median (IQR)** | **Version A-Summary % Scores**  **Q1, Q2, Q4, Q5 >4** **Q3 ≤2** | **Version B-Full report Median (IQR)** | **Version B-Full report % Scores** **Q1, Q2, Q4, Q5 >4** **Q3 ≤2** | **Median difference (B-A)** | **Wilcoxon W (B-A)** | ***P*-value (B-A)** |
| --- | --- | --- | --- | --- | --- | --- | --- |
| Question 1: Version is factually correct? | 4.670 (4.330 - 5.000) | 80% | 4.330 (4.000 - 4.670) | 67% | -0.335 | -241.000 | 0.001 |
| Question 2: Version includes relevant clinical information? | 4.670 (4.670 - 5.000) | 97% | 4.500 (4.330 - 4.670) | 90% | -0.330 | -179.000 | <0.0001 |
| Question 3: Version includes harmful information, which might result in physical and/or psychological harm? | 1.670 (1.670 - 2.000) | 83% | 2.000 (2.000 - 2.330) | 73% | 0.330 | 192.000 | 0.001 |
| Question 4: Version is accessible and readable for a patient/lay person? | 4.670 (4.670 - 5.000) | 97% | 4.670 (4.330 - 4.670) | 83% | -0.330 | -116.000 | 0.026 |
| Question 5: Version is beneficial to patients, in addition to the original report? | 4.670 (4.330 - 5.000) | 87% | 4.000 (3.670 - 4.000) | 17% | -0.670 | -435.000 | <0.0001 |

Table 1: Radiologist Lung - Median scores across readers and Wilcoxon tests

| **Question** | **Version A-Summary Median (IQR)** | **Version A-Summary % Scores**  **Q1, Q2, Q4, Q5 >4** **Q3 ≤2** | **Version B-Full report Median (IQR)** | **Version B-Full report % Scores** **Q1, Q2, Q4, Q5 >4** **Q3 ≤2** | **Median difference (B-A)** | **Wilcoxon W (B-A)** | ***P* -value (B-A)** |
| --- | --- | --- | --- | --- | --- | --- | --- |
| Question 1: Version is factually correct? | 4.165 (3.330 -4.330) | 50% | 4.670 (4.000 - 4.670) | 73% | 0.340 | 247.000 | 0.002 |
| Question 2: Version includes relevant clinical information? | 4.000 (3.670 - 4.330) | 47% | 4.670 (4.585-4.670) | 83% | 0.505 | 290.000 | <0.0001 |
| Question 3: Version includes harmful information, which might result in physical and/or psychological harm? | 1.670 (1.330 - 2.330) | 70% | 1.330 (1.330 - 1.670) | 90% | -0.330 | -154.000 | 0.001 |
| Question 4: Version is accessible and readable for a patient/lay person? | 4.000 (4.000 - 4.330) | 47% | 4.330 (4.248 - 4.670) | 77% | 0.330 | 197.000 | 0.001 |
| Question 5: Version is beneficial to patients, in addition to the original report? | 3.670 (3.248 - 4.000) | 3% | 4.000 (3.670 - 4.330) | 43% | 0.340 | 369.000 | <0.0001 |

Table 2: Radiologist Colorectal - Median scores across readers and Wilcoxon tests

| **Question** | **Version** | **Medium (IQR) First read** | **Medium (IQR) Re-read** | ***P* -value (A - First read versus Re-read)** | ***P* -value (B - First read versus Re-read)** |
| --- | --- | --- | --- | --- | --- |
| Question 1: Version is factually correct? | A | 4.833 (4.250 - 5.000) | 4.667 (4.333 - 4.750) | >0.9999 | 0.625 |
|  | B | 4.333 (4.250 - 4.667) | 4.333 (4.000 - 4.417) |  |  |
| Question 2: Version includes relevant clinical information? | A | 5.000 (4.583 - 5.000) | 3.500 (3.333 - 4.500) | 0.063 | 0.156 |
|  | B | 4.667 (4.250 - 4.667) | 3.833 (3.667 - 4.417) |  |  |
| Question 3: Version includes harmful information, which might result in physical and/or psychological harm? | A | 1.833 (1.667 - 2.417) | 1.667 (1.583 - 2.083) | 0.250 | >0.9999 |
|  | B | 2.000 (1.917 - 2.167) | 2.000 (1.667 - 2.333) |  |  |
| Question 4: Version is accessible and readable for a patient/lay person? | A | 4.667 (4.333 - 5.000) | 4.667 (4.583 - 4.750) | >0.9999 | 0.063 |
|  | B | 4.667 (4.500 - 4.667) | 4.333 (4.250 - 4.333) |  |  |
| Question 5: Version is beneficial to patients, in addition to the original report? | A | 4.500 (4.333 - 5.00) | 4.667 (4.583 - 4.750) | 0.688 | 0.063 |
|  | B | 4.000 (3.833 - 4.083) | 4.333 (4.250 - 4.333) |  |  |

Table 3: Radiologist Lung - Repeatability

| **Question** | **Version** | **Medium (IQR) First read** | **Medium (IQR) Re-read** | ***P* -value (A - First read versus Re-read)** | ***P* -value (B - First read versus Re-read)** |
| --- | --- | --- | --- | --- | --- |
| Question 1: Version is factually correct? | A | 4.000 (3.500-4.417) | 4.667 (3.500-5.000) | 0.2812 | 0.2812 |
|  | B | 4.667 (4.083-4.667) | 4.833 (4.333-5.000) |  |  |
| Question 2: Version includes relevant clinical information? | A | 4.000 (3.500-4.417) | 4.667 (4.250-4.667) | 0.125 | 0.375 |
|  | B | 4.667 (4.250-4.667) | 4.667 (4.583-5.000) |  |  |
| Question 3: Version includes harmful information, which might result in physical and/or psychological harm? | A | 1.833 (1.333-2.750) | 1.333 ( 1.333-1.750) | 0.25 | 0.125 |
|  | B | 1.667 (1.333-1.750) | 1.333 (1.333-1.333) |  |  |
| Question 4: Version is accessible and readable for a patient/lay person? | A | 4.167 (3.583-4.333) | 4.333 (4.250-4.417) | 0.125 | 0.125 |
|  | B | 4.333 (4.000-4.667) | 4.667 (4.667-4.667) |  |  |
| Question 5: Version is beneficial to patients, in addition to the original report? | A | 3.500 (3.167-3.750) | 4.000 (3.500-4.333) | 0.3125 | 0.0625 |
|  | B | 4.000 (3.667-4.333) | 4.333 (4.250-4.667) |  |  |

Table 4: Radiologist Colorectal - Repeatability

| **Question** | **Original version Median (IQR)** | **Version A-Summary Median (IQR)** | **Version B-Full report Median (IQR)** | **Friedman X2** | **p-value (overall)** | **Median difference (B-A)** | **Wilcoxon W (B-A)** | | ***P* -value (B-A)** |
| --- | --- | --- | --- | --- | --- | --- | --- | --- | --- |
| Q1: Accessibility and readability for a patient/lay person | 1.667  (1.583 - 1.667) | 4.000  (3.583- 4.333) | 4.333  (4.333 - 4.417) | 17.68 | <0.0001 | 0.667 | 29 | 0.047 | |
| Q2: Beneficial to patients | 2.833  (2.667 - 3.333) | 3.500  (3.167 - 3.667) | 4.000  (3.667 - 4.083) | 17.21 | <0.0001 | 0.333 | 45.000 | 0.0039 | |
| Q3: Would like to have access to that version of the report | 1.333  (1.333 - 1.333) | 1.333  (1.333 - 1.417) | 1.000  (1.000 - 1.333) | 11.840 | 0.0018 | -0.333 | -28.000 | 0.016 | |

Table 5: PPI Lung - Median scores across reader, Friedman and Wilcoxon tests

| **Question** | **Original version Median (IQR)** | **Version A-Summary Median (IQR)** | **Version B-Full report Median (IQR)** | **Friedman X2** | ***P* -value (overall)** | **Median difference (B-A)** | **Wilcoxon W (B-A)** | ***P* -value (B-A)** |
| --- | --- | --- | --- | --- | --- | --- | --- | --- |
| Q1: Accessibility and readability for a patient/lay person | 1.667  (1.333 - 1.667) | 4.333  (3.667 - 4.667) | 4.333  (4.250 - 4.417) | 15.790 | <0.0001 | 0.000 | 10.000 | 0.570 |
| Q2: Beneficial to patients | 3.167  (2.333 - 3.333) | 3.667  (3.333 - 3.833) | 4.000  (3.667 - 4.000) | 13.470 | 0.000 | 0.333 | 22.000 | 0.270 |
| Q3: Would like to have access to that version of the report | 1.333  (1.333 - 1.417) | 1.333  (1.333 - 1.333) | 1.000  (1.000 - 1.083) | 14.970 | 0.000 | -0.333 | -45.000 | 0.004 |

Table 6: PPI Colorectal - Median scores across readers, Friedman and Wilcoxon tests
