# Supplementary material for: Improving patient understanding of oncology imaging: radiologist and patient evaluation of summarised versus full-length AI-simplified reports from a tertiary cancer centre
Source: Cancer Imaging. 2026 Apr 13;26:68. doi: 10.1186/s40644-026-01031-x (PMC13188515; doi:10.1186/s40644-026-01031-x)
Supplement: Supplementary file 4 — Supplementary Material 4 [file 40644_2026_1031_MOESM4_ESM.docx]

# Additional file 4. Figures

Figure 1: Radiologist Lung - Median scores for relevant factual correctness

Figure 2: Radiologist Lung - Median scores for relevant clinical information

Figure 3: Radiologist Lung - Median scores for introduction of harmful information

Figure 4: Radiologist Lung - Median scores for accessibility and readability

Figure 5: Radiologist Lung - Median scores for patient benefit

Figure 6: Radiologist Colorectal - Median scores for factual correctness

Figure 7: Radiologist Colorectal - Median scores for relevant clinical information

Figure 8: Radiologist Colorectal - Median scores for introduction of harmful information

Figure 9: Radiologist Colorectal - Median scores for accessibility and readability

Figure 10: Radiologist Colorectal - Median scores for patient benefit

Figure 11: PPI Lung - Median scores for accessibility and readability

Figure 12: PPI Lung - Median scores for benefit to patients

Figure 13: PPI Lung - Median scores for access to version of report

Figure 14: PPI Colorectal - Median scores for accessibility and readability

Figure 15: PPI Colorectal - Median scores for benefit to patients

Figure 16: PPI Colorectal - Median scores for access to version of report

Figure 17: PPI Colorectal & Lung - Median scores across readers for accessibility and readability

Figure 18: PPI Colorectal & Lung - Median scores across readers for benefit to patients

Figure 19: PPI Colorectal & Lung - Median scores across readers for access to version of report
